# Managed Activity Graded Exercise iN Teenagers and pre-Adolescents (MAGENTA) feasibility randomised controlled trial: study protocol

**DOI:** 10.1136/bmjopen-2016-011255

**Published:** 2016-07-04

**Authors:** Amberly Brigden, Lucy Beasant, William Hollingworth, Chris Metcalfe, Daisy Gaunt, Nicola Mills, Russell Jago, Esther Crawley

**Affiliations:** 1 School of Social and Community Medicine, University of Bristol, Bristol, UK; 2 Bristol Randomised Trials Collaboration & School of Social and Community Medicine, University of Bristol, Bristol, UK; 3 Centre for Exercise, Nutrition & Health Sciences, School for Policy Studies, Bristol, UK

**Keywords:** PAEDIATRICS, chronic fatigue syndrome, CFS/ME, ME, GET

## Abstract

**Introduction:**

Paediatric chronic fatigue syndrome or myalgic encephalomyelitis (CFS/ME) is a relatively common and disabling condition, yet there is a limited evidence base for treatment. There is good evidence that graded exercise therapy is moderately effective in adults with CFS/ME, but there is little evidence for the effectiveness, cost-effectiveness, acceptability or best method of delivery for paediatric CFS/ME. This study aims to investigate the acceptability and feasibility of carrying out a multicentre randomised controlled trial investigating the effectiveness of graded exercise therapy compared with activity management for children/teenagers who are mildly or moderately affected with CFS/ME.

**Methods and analysis:**

100 paediatric patients (8–17 years) with CFS/ME will be recruited from 3 specialist UK National Health Service (NHS) CFS/ME services (Bath, Cambridge and Newcastle). Patients will be randomised (1:1) to receive either graded exercise therapy or activity management. Feasibility analysis will include the number of young people eligible, approached and consented to the trial; attrition rate and treatment adherence; questionnaire and accelerometer completion rates. Integrated qualitative methods will ascertain perceptions of feasibility and acceptability of recruitment, randomisation and the interventions. All adverse events will be monitored to assess the safety of the trial.

**Ethics and dissemination:**

The trial has received ethical approval from the National Research Ethics Service (South West—Frenchay 15/SW/0124).

**Trial registration number:**

ISRCTN23962803; Pre-results.

**Strengths and limitations of this study**

- This feasibility study is the first trial to investigate graded exercise therapy in children with chronic fatigue syndrome or myalgic encephalomyelitis (CFS/ME) in the outpatient setting.
- Integrated qualitative methodology is being used to optimise recruitment and retention, and to investigate the feasibility and acceptability of the study processes and interventions.
- This is a multicentre study which will test the feasibility of running this trial in different National Health Service (NHS) settings.
- The participants and clinicians will not be blinded to allocation.
- Participant outcomes are self-reported.

## Introduction

Chronic fatigue syndrome or myalgic encephalomyelitis (CFS/ME) in children is relatively common, affecting between 0.1% and 2% of secondary school-aged children.^1–4^ CFS/ME is defined as ‘generalised fatigue, causing disruption of daily life, persisting after routine tests and investigations have failed to identify an obvious underlying “cause”’.^5^ ^6^ National Institute for Health and Care Excellence (NICE) guidelines recommend a minimum 3-month duration of fatigue before making a diagnosis in children.^5^

NICE recommends that children with CFS/ME are offered either cognitive–behavioural therapy (CBT), graded exercise therapy (GET) or activity management.^5^ GET stabilises physical activity levels, before gradually increasing at a manageable rate.^5^ ^7–11^ Activity management establishes a baseline for all the activities (mainly cognitive, such as school and homework, in children and adolescents), which is then increased.^5^ ^12^ There is good evidence for the effectiveness of CBT in children with CFS/ME;^13–15^ however, there is little evidence for the effectiveness of GET in children and adolescents, although GET is moderately effective in adults.^7^ There is also limited evidence on the acceptability of GET for children and adolescents with CFS/ME or on the best method for delivering these interventions in terms of intensity (frequency of sessions) and length of interventions (number of sessions and length of time for interventions).

In addition to estimating study parameters such as the willingness of participants to be randomised and the number of eligible patients,^16^ feasibility studies can be used to improve recruitment and retention by audio-recording recruitment discussions, evaluating information exchange and training those who recruit to the study.^17^ ^18^ Integrated qualitative methodology can also be used to investigate participants' view of interventions and study methodology, providing an opportunity to improve them prior to the full trial.^19^

In this study, we will determine whether it is acceptable and feasible to deliver GET compared with activity management in a multicentre randomised controlled trial (RCT). The trial is designed as a pragmatic trial as we are interested in the effectiveness of interventions delivered in routine practice.^20^ Integrated qualitative methods will be used to optimise recruitment retention and the delivery of the interventions, and to investigate the best method for measuring outcomes.

## Aims and objectives

To ascertain the feasibility and acceptability of conducting an RCT to investigate the effectiveness and cost-effectiveness of GET compared with activity management for the treatment of CFS/ME in children. We will use the information to inform the design of a full-scale, adequately powered trial. The specific objectives are to:

1. Assess the number of eligible children and adolescents, the number of children and adolescents approached, the number recruited and the number retained in the first 6 months of the study.
2. Identify barriers and facilitators to trial recruitment with a view to addressing barriers where possible.
3. Explore issues of retention and understand why people drop out of the study.
4. Assess the acceptability (satisfaction and adherence) of GET and activity management.
5. Assess the feasibility and acceptability of using accelerometers to measure physical activity in children and adolescents with CFS/ME.
6. Evaluate whether the two interventions are distinct and being delivered in a consistent manner across centres.

## Method

### Study design

This study started in September 2015 and recruitment is expected to finish in August 2016. This is a feasibility RCT with integrated qualitative methods.

### Participants and recruitment

Potential participants will be identified by the clinician conducting the first assessment at specialist paediatric CFS/ME services in Bath, Newcastle and Cambridge. The clinician will provide information about the study and obtain written assent/consent for a member of the research team to talk to the child and parent/carers about the study and for this discussion to be recorded.

Potential participants can either meet the recruiting researcher in the hospital on the day of the initial assessment or discuss the study at a later date on the phone. At the start of the recruitment to trial discussion, the recruiter will confirm assent/consent for the discussion and check that the parent continues to be happy to have the discussion audio-recorded. The recruiting researcher will then discuss and provide further information about the Managed Activity Graded Exercise iN Teenagers and pre-Adolescents (MAGENTA) trial, including the study design, interventions, participant burden and the potential risks and benefits of taking part.

Children and parents who wish to take part in the study can either complete the written study assent/consent forms with the recruiter at the end of the discussion, returning them via post, scan the forms and email them electronically or sign the web-based consent form provided through the University of Bristol's data capture system (Research Electronic Data Capture (REDCap), http://project-redcap.org/). A key aim of the feasibility trial is to ascertain the usefulness of the consent procedures.

### Inclusion/exclusion criteria

Children and adolescents will be eligible for inclusion if they are given a diagnosis of CFS/ME (made using NICE guidance^5^) and aged between 8 and 17 years inclusive.

Children and adolescents will be excluded if they are severely affected. NICE defines severe CFS/ME as individuals who are unable to do activity for themselves, or carry out minimal daily tasks only, have severe cognitive difficulties and depend on wheelchair for mobility;^5^ or are referred for CBT at their first clinical assessment; or are unable to attend clinical sessions. Eligibility assessment will be carried out by the clinician at assessment and confirmed by the recruiting researcher.

### Randomisation and allocation

Once the recruiter has received the signed assent/consent forms, they will use the automated telephone/web randomisation service operated by the Bristol Randomised Trials Collaboration. Allocation (1:1) will use minimisation to facilitate balance by age and gender, and retain a random component to prevent accurate prediction of allocation. Owing to the nature of the interventions, it is not practical to keep either the family or the clinical service blind to intervention allocation. If allocation is done during the recruitment appointment, families can choose to know the allocation immediately or be told later by phone or letter (if told via phone, the discussion will be audio-recorded with assent/consent from the parent/child). The recruiter then informs the clinical service who writes to the parent/child with their appointment details. The child's general practitioners (GPs) will be told what intervention they will receive as part of routine clinical practice.

### Sample size

An estimated 380 children and adolescents are assessed per annum in the three centres (Bath/Bristol 300, Cambridge 50, Newcastle 30). Estimates based on the Specialist Medical Intervention and Lightning Evaluation (SMILE) trial^12^ suggest 60% will be eligible, of which 40–50% will be recruited. We therefore estimate that recruitment of 100 will take ∼12 months.

Recruiting 100 children from 430 eligible children approached will give a 95% CI of 20% to 28% for an estimated recruitment rate of 24% (0.6 eligible×0.4 consenting), which is acceptably precise for planning the main study recruitment.

### Clinical interventions

Both interventions will be delivered in an outpatient setting. During clinical sessions, clinicians and patients develop collaborative activity plans, which children and adolescents then implement in the community (including home and school environments). Children and adolescents will be advised to use paper diaries/apps to assist with monitoring and recording of activity levels. In both arms, children and adolescents, their parents and the clinician providing intervention will choose the number of clinical sessions (between 8 and 12) and the frequency of appointments (every 2–6 weeks) within a maximum length of treatment of 1 year.

In both arms, clinicians will be encouraged to offer routine^5^ advice about sleep, medication use, symptom control and setbacks at the assessment and during intervention sessions.

Participants who develop anxiety or depression that requires treatment during the trial follow-up period will be offered up to 12 sessions of CBT delivered as individual sessions every 2 weeks by a CFS/ME specialist clinical psychologist.

Participants will be allowed to either discontinue the intervention or withdraw from the trial at any time. If parents or clinicians request cross-over to the other intervention arm, we will encourage them to try the original allocation for 6 months (the primary outcome). Any cross-over will be recorded.

#### Arm 1: activity management

This arm will be delivered by CFS/ME specialists. As activity management is currently being delivered in all three services, clinicians will not require further training; however, they will receive guidance on the mandatory, prohibited and flexible components (see online supplementary appendix 1). Clinicians therefore have flexibility in delivering the intervention within their National Health Service (NHS) setting. Activity management aims to convert a ‘boom–bust’ pattern of activity (lots 1 day and little the next) to a baseline with the same daily amount before increasing the daily amount by 10–20% each week. For children and adolescents with CFS/ME, these are mostly cognitive activities: school, schoolwork, reading, socialising and screen time (phone, laptop, TV, games). Those allocated to this arm will receive advice about the total amount of daily activity, including physical activity, but will not receive specific advice about their use of exercise, increasing exercise or timed physical exercise.

10.1136/bmjopen-2016-011255.supp1 supplementary appendix

#### Arm 2: graded exercise therapy (GET)

This arm will be delivered by referral to a GET-trained CFS/ME specialist who will receive guidance on the mandatory, prohibited and flexible components (see online supplementary appendix 1). They will be encouraged to deliver GET as they would in their NHS setting.^20^ Those allocated to this arm will be offered advice that is focused on exercise with detailed assessment of current physical activity, advice about exercise and a programme including timed daily exercise. The intervention will encourage children and adolescents to find a baseline level of exercise which will be increased slowly (by 10–20% a week, as per NICE guidance^5^ and the Pacing, graded Activity and Cognitive behaviour therapy – a randomised Evaluation (PACE)^12^ ^21^). This will be the median amount of daily exercise done during the week. Children and adolescents will also be taught to use a heart rate monitor to avoid overexertion. Participants will be advised to stay within the target heart rate zones of 50–70% of their maximum heart rate.^5^ ^7^

For further details about both intervention arms, please see online supplementary appendix 1.

### Integrated qualitative methods

Qualitative research methods will be integrated into the feasibility study to optimise the recruitment process and investigate acceptability of the interventions and wider trial processes.

#### Trial processes

The recruiting researcher will receive a training session on the two interventions and will be encouraged to provide a balanced description of each (by spending an equal amount of time discussing the benefits and drawbacks of both intervention arms). To identify recruitment difficulties and improve recruitment,^18^ we will audio-record (with assent/consent) recruitment discussions. A sample of these will be analysed at regular intervals to explore information provision, recruitment techniques, patient intervention preferences and trial participation decisions. If analyses of the audio-recordings identify issues that appear to be impacting on trial recruitment, then feedback and training will be offered to the recruiter, the content of which will depend on the findings.

If the number of eligible patients recruited is lower than expected, or if there are differences in the percentage recruited between centres, we may undertake in-depth interviews with the clinical and recruitment staff and analyse screening logs to examine possible problems with patient pathways in the different centres.

We will undertake in-depth interviews with parents and their children and adolescents to understand their views and experiences of trial processes. This will include provision and acceptability of patient information and reasons for accepting or declining participation. We are particularly interested in understanding barriers to participation and with assent/consent will interview those who choose not to participate in the trial, who drop out of trial follow-up or who do not accept intervention allocation at randomisation. We will interview children and adolescents and their parents about their use of the accelerometer, whether it is an acceptable device to wear and whether there are particular issues we need to consider in this patient group for the full trial.

#### Trial interventions

Intervention sessions will be routinely audio-recorded, with consent, to enable us to test that activity management and GET are truly distinct, and to ensure that the interventions are delivered in a consistent manner across centres. The delivery of up to 10 interventions, in both arms in each centre, will be observed. Sessions delivered early in the feasibility study will be observed initially, with further sessions being sampled depending on the analysis of these initial observations. Detailed notes will be taken, including the context, intensity and variability of intervention delivery, to understand how interventions are delivered and received in practice and to help interpret outcomes (eg, variation between subgroups).

We will interview parents and their children and adolescents about both interventions, including any prior exposure to the study interventions; beliefs, expectations and preferences about the interventions before assignment; their experiences and acceptability of the interventions; use of heart rate monitors (including whether they increase or decrease anxiety); and their views of the number of intervention sessions required. Participants will be recruited from all three centres to assess differences in implementation between settings.

We will interview clinicians delivering both interventions in each centre to ascertain their views on the feasibility of delivery (particularly focusing on younger children), changes that need to be made to the interventions offered, optimal frequency of appointments and technical problems with using heart rate monitors.

Interviews with clinicians, participants and families will be semistructured using a topic guide to ensure that they cover the same issues while allowing new issues of importance to emerge. Sampling for interviews will ensure that a range of informants (in terms of age, gender, ethnicity, geographical location, socioeconomic circumstances and disease severity) are included (maximum variation sampling), and that people with particular characteristics of interest can be targeted to follow-up and develop emerging findings (theoretical sampling). Sample size will be determined by data saturation, that is, when no new themes are being uncovered.^22^ It is anticipated that up to 20 patients, 15 parents and 10 clinicians involved in recruitment and/or delivering the interventions will be interviewed at a location of their choice. We estimate that up to 45 patient, parent and clinical staff interviews will be sufficient to determine whether it is feasible and acceptable to take the study to full trial, and to identify ways to improve study processes. All discussions will be audio-recorded with assent/consent using encrypted software, transcribed verbatim and anonymised.

### Outcomes measures

#### Feasibility outcomes measurement

We will use quantitative and qualitative data to determine the feasibility and acceptability of a full-scale multicentre RCT. Findings will be fed back to the research team to improve the design, conduct and organisation of the main trial.

Quantitative data will include the number of children and adolescents who were eligible, approached, consented and retained in the study; the completeness of questionnaire data at baseline assessment and follow-up; the percentage of children and adolescents providing usable accelerometer data; and the proportion of participants who found their allocated intervention acceptable and adhered to the intervention programme (proportion of planned sessions attended) in each arm. The number of participants in each arm referred for CBT will be ascertained from hospital records.

#### Quantitative analysis

The percentage recruited of those eligible will be calculated from the screening log data and presented with 95% CI. Retention will be estimated as the percentage of recruited children and adolescents reaching the primary 6-month follow-up point, who provide key outcome measures (the Chalder Fatigue Scale and the 36-Item Short-Form Physical Functioning Scale (SF-36 PFS)) at that assessment point. The retention estimates will be presented for each intervention arm with 95% CIs. We will record the number of booked intervention sessions where participants did not attend or where there was a late cancellation (within 24 hours). We will assume that those who did not attend (or cancelled within 24 hours) three or more consecutive appointments or 50% of appointments did not find the interventions acceptable.

We will calculate the proportion of children and adolescents who wear the accelerometer and provide usable data. We will assume periods of 60 min or more with zero readings as ‘non-wear’ time. Participant's data will be included if they provide two or more weekdays of data with at least 500 min of data between 6:00 and 23:00. Mean minutes of weekday, light and moderate-to-vigorous physical activity (MVPA) per day will be established using the Evenson thresholds,^23^ which have been shown to be the most accurate for this age group.

We will collect the number, frequency and length of clinical sessions for each participant.

At 12 months, we will assess whether the trial should continue to a full trial. The full trial is unlikely to be feasible in the format considered here if any of the following apply:

1. Less than 70 children and adolescents have been recruited (∼70% of the target) and if the qualitative data collected suggest that recruitment cannot be improved any further.
2. The 6-month follow-up is <80% and if the qualitative data suggest that follow-up rates cannot be improved any further.
3. Data suggest the interventions are not acceptable to children and/or their parents.
4. If the Data and Safety Monitoring Committee (DSMC) and the Trial Steering Committee (TSC) recommend the trial is stopped for safety reasons.

*Fidelity of GET and activity management*: we will monitor protocol adherence and evaluate whether the two interventions are distinct and being delivered in a consistent manner across centres. Two clinicians, from centres other than that in which the session was delivered, will listen to a random sample (∼10%) of the audio-recorded sessions in a blinded fashion and rate them on a five-point Likert scale as being GET or activity management or a mixture of the two using the mandatory, prohibited and flexible elements for each intervention. We will estimate intervention fidelity as the percentage of sessions in each intervention arm which were correctly identified by the clinicians assessing recordings.

*Health economic measures*: we will assess the feasibility of using routine data to gather information on the initial costs of the GET and activity management interventions and other specialist services (eg, CBT) offered to children and adolescents. We will test the acceptability of collecting healthcare resource use data at 6 and 12 months to estimate the other CFS/ME-related costs to the NHS, other government agencies and the broader impact on family expenses, productivity and informal care.

*Safety* *outcomes:* we will collect all serious and non-serious adverse events defined as any clinical change or illness reported at clinic or postal follow-up. In addition, we will define a serious deterioration in health as a decrease ≥20 in SF-36 PFS or scores of ‘much’ or ‘very much worse’ on the Clinical Global Impression Scale; clinician-reported serious deterioration in health; or withdrawal from intervention because of feeling worse. Safety outcomes will be analysed by the DSMC at 10 months to ensure that neither intervention arm is having a detrimental effect. The DSMC will include three independent experts in CFS/ME, statistics and trials and will report to the TSC.

#### Clinical outcome measures

Patient-reported outcomes will be collected at baseline, 6 and 12 months postrandomisation (see table 1).

**Table 1:** Data routinely collected at assessment. Questionnaire data collected at baseline, 6 and 12 months

Assessment data	Questionnaires
Age	Chalder fatigue^24^
Sex	Physical function (SF-36)^25^
Ethnicity (drop-down list)	Hospital Anxiety Depression Scale^26^
School attendance	Spence Children's Anxiety Scale^27^
Symptoms list	Pain Visual Analogue Scale
CDC and NICE criteria	Quality of life (EQ-5D-Y^28^)
Months of illness	Clinical Global Impression Scale*
Comorbid conditions	

*Collected at 6-month and 12-month follow-up only.

CDC, Centers for Disease Control; NICE, National Institute for Health and Care Excellence.

Both parents will be asked to complete three inventories online at baseline, 6 and 12-month follow-up, including socioeconomic status (baseline only); an adapted four-item Work Productivity and Activity Impairment Questionnaire (General Health V2.0 [WPAI:GH]);^29^ and an adapted existing healthcare resource use questionnaire to measure health service use (eg, GP or specialist care), educational service (eg, school counsellor) and travel costs. The acceptability of these inventories in this participant group has been tested.^19^ Information from the specialist services medical records will be extracted to identify referrals for additional CBT or referrals to Child and Adolescent Mental Health Services within 12 months of randomisation.

If questionnaires are not returned, an email reminder (or postal reminder if the participant does not have internet access) will be sent after 1 week. If outcomes are still not completed, a further email (or postal) reminder will be sent with a link to a reduced number of questions containing just the Chalder Fatigue Scale, the SF 36 PFS and the EQ-5D-Y as these were shown to be acceptable in our previous trial.^19^ If this is not completed, we will make up to four follow-up telephones calls or emails and offer to collect the primary outcome data over the phone.

**Figure 1:**
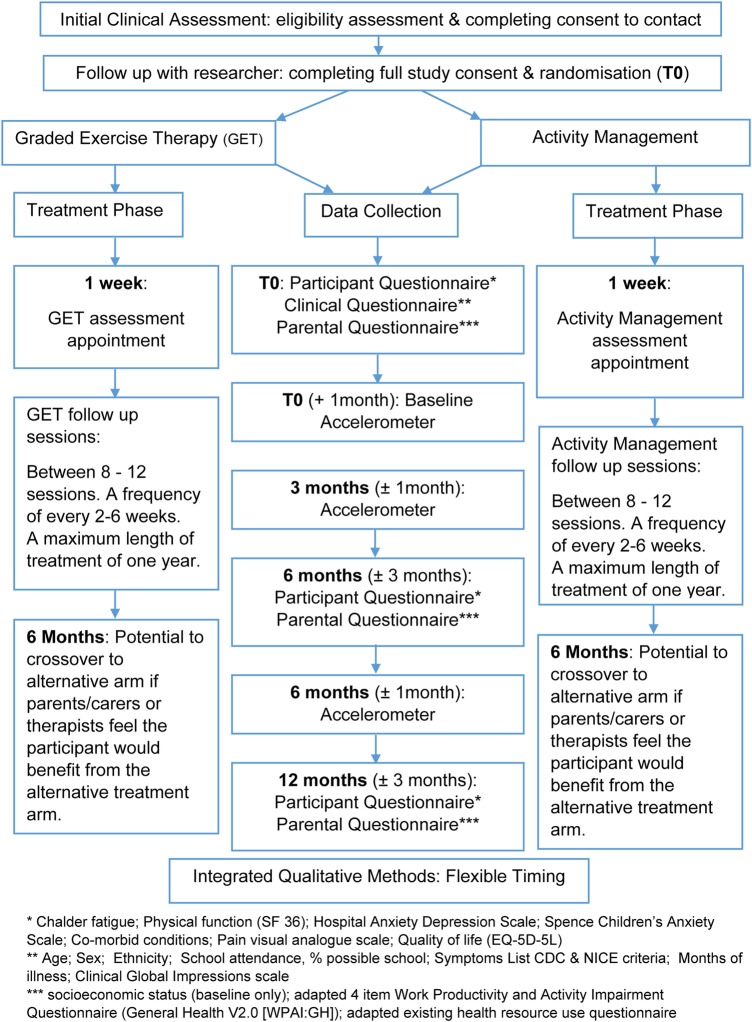
Study flow diagram detailing participant flow through the study-related interventions and data collection procedures. CDC, Centers for Disease Control; NICE, National Institute for Health and Care Excellence.

In addition to questionnaire measures, participants in both trial arms will be asked to wear an accelerometer (GT3X+) to measure physical activity for 7 days within 1 month of randomisation and at 3 and 6-month follow-up. During this 7-day period, participants will be instructed to wear the device for the entirety of the day. Accelerometers will be posted to participants with instructions. Participants will be asked to complete a log of wear time (time worn and time taken off). Accelerometers are small, matchbox-sized devices that measure physical activity. They have been shown to provide reliable indicators of physical activity among children and adults.^30^ The accelerometer data will be processed to identify mean minutes of sedentary, light and moderate-to-vigorous-intensity physical activity per day using established accelerometer cut-off points and protocols.^23^ ^31^ The mean accelerometer counts per minute, which provides an indication of the volume of physical activity in which the participant engages, will also be calculated using established methods.^7^ ^23^ See figure 1 for the study flow diagram.

## Qualitative data analyses

Analysis of the audio-recordings, observations and interviews will be an ongoing and iterative process commencing soon after data collection. Emerging findings will inform further sampling and data collection. A multiple analytical approach will be employed, with the use of NVivo to assist with data management and analysis. Interview transcripts and observation notes will be systematically assigned codes and analysed thematically using techniques of constant comparison to identify common themes (thematic analysis). Individuals exhibiting contrasting attitudes (negative cases) will be studied in detail. The perspectives of the individuals will be paramount, with careful account taken of the context within which the discussion takes place.

Content analytic methods will be used to describe in a structured manner what was said by whom and how often in the audio-recordings of recruitment and intervention sessions. Thematic analysis will also be applied to the data to identify common or divergent themes, as described above, particularly focusing on the impact of statements by the recruiter/clinician on patients and parents. Conversation analysis will be used to focus in great detail on certain sections of the audio-recordings, for example, in the specific interactions during which randomisation is offered.

Data analyses will be undertaken primarily by the qualitative researcher. To check coding reliability, other members of the team will independently analyse a proportion of transcripts and compare findings.

## Ethics and dissemination

### Ethical considerations

GET, CBT and activity management are recommended as treatments in NICE guidance for CFS/ME;^5^ however, there is no evidence that GET is effective or safe in children and adolescents. CFS/ME is different in children and adults with different risk factors, course and outcome.^32^ It is therefore not possible to extrapolate the results from adult studies to children. A trial in children is therefore required.

At the moment, CBT has the best evidence for treatment efficacy. We have not included CBT as the control arm because clinicians would not be in equipoise in randomising children to CBT or GET. CBT will continue to be offered to young people who develop mood problems after randomisation and can be accessed in addition to either treatment arm. We will analyse referral to CBT as a secondary outcome.

As the participants will be aged 8–17 years, we have put in place rigorous procedures for gaining informed assent/consent from children and adolescents and their parents. Families will be given as long as they need before giving assent/consent within the confines of the study. We will then obtain further assent/consent prior to each interview and prior to recording interventions to check that children and adolescents and their parents continue to be willing to participate.

#### Data protection/confidentiality

Participants will be allocated a unique seven-digit research identification number prior to assessment for screening logs and questionnaires. Assent/consent forms containing personal information will be kept in a locked filing cabinet in a locked office within the University of Bristol. A list of names and corresponding identification numbers will be kept separately and securely on a password-protected NHS server.

Data will be entered into REDCap, a secure system used by multiple institutions for large multicentre studies. Assessment data will be entered by the research team. Participants will be encouraged to provide follow-up data through REDCap using a secure password-protected login, but will be able to provide data by post if they do not have internet access.

#### Dissemination of research findings

Research findings will be disseminated through peer-reviewed research journals, conferences and patient organisations.

#### Ethics approval

The trial has been reviewed and granted approval by the National Research Ethics Service Committee (South West—Frenchay 15/SW/0124).

#### Public and patient involvement

A Patient Advisory Group (PAG) has been involved throughout the development of this protocol and will remain involved throughout the running of the trial, with PAG meetings being held every 6 months. Minutes from the PAG group will be disseminated to the Study Steering Committee and the Trial Management Group.
