# Supplementary material for: Managed Activity Graded Exercise iN Teenagers and pre-Adolescents (MAGENTA) feasibility randomised controlled trial: study protocol
Source: BMJ Open. 2016 Jul 4;6(7):e011255. doi: 10.1136/bmjopen-2016-011255 (PMC4947787; doi:10.1136/bmjopen-2016-011255)
Supplement: supplementary appendix [file bmjopen-2016-011255supp_appendix.pdf]

Mandatory, flexible and prohibited elements of both trial arms (Graded Exercise Therapy and Activity Management)

Arm 1: Activity Management

Mandatory elements: Therapists will discuss the different types of cognitive activity (high concentration and low concentration) which will vary according to age. Participants will be taught how to find their baseline of cognitive activities. Cognitive activities include time at school or doing school work, reading, some craft/hobbies, socialising and screen time (phone, laptop, TV, computer, other devices). The baseline is the median time spent doing cognitive activity and can either be estimated in collaboration with the specialist therapist or calculated after a period of recording activity. Once the baseline is agreed with participants, they will be asked to record the total number of minutes spent each day doing high-energy cognitive activities using paper diaries or our award-winning smartphone app “ActiveME”. Recording activity is used to help participants understand whether they are doing the same each day or varying their activity and whether the baseline has been set at the correct level. When participants have managed the baseline for 1-2 weeks, they will be asked to increase this by 10-20% each week[5]. Therapists will discuss problems encountered by participants and provide possible solutions.

Managing setbacks will be discussed (how much to reduce school and other cognitive activity and for how long). Participants will continue to increase activity until they are able to do at least 8 hours of cognitive activity a day.

Prohibited*:* Discussion about number of steps, minutes of exercise, aerobic, versus non aerobic activity. No discussion about increasing physical activity (only discussion about increasing overall activity). No advice on exercises or using a strengthening programme.

Flexible*:* Advice on Physical Education (PE) in school (no PE, half a lesson, full lesson). Attendance at sporting events (do not attend, attend limited period of time). Children and young people can record physical activity within the total cognitive activity but are not required to do so.

Arm 2: Graded Exercise Therapy

Mandatory*:* Physical assessment, assessment of range and type of exercise used during the week at the first assessment. Functional muscle test at assessment and 6 months including: sit to stand and 2 minute walk test (distance covered). Exercise targets will be negotiated with the child and parents/carer. Initial exercise targets (the baseline) will be the median amount of daily exercise done during the week. Once this is achieved every day for one to two weeks, participants will be advised to increase exercise slowly by 10-20% a week. They will be asked to time their exercise to make sure they are completing the same number of minutes of exercise every day and record these minutes of exercise each day using either paper diaries or our smartphone app "ActiveME". Diaries (paper or using ActiveME) will be reviewed to help children ensure their exercise is the same every day. Once participants are doing 30 minutes of planned exercise within the low intensity heart rate limits (such as slow paced walking), the exercise will increase in intensity such that participants start doing exercise that increases their heart rate (for example faster paced walking, cycling).

The exercise programme will be negotiated and agreed together at each appointment between the therapist, child and adolescent and parent/carer.

Participants aged 10 and over will be taught how to monitor their heart rate using a heart rate monitor to prevent them doing too high an exercise intensity. They will be set a target heart rate and asked not to exceed this[5 7]. Younger children or those who cannot measure their heart rate will learn how to monitor their heart rate with their parents.

Managing setbacks will be discussed prior to discharge in the context of physical exercise (how much this should be reduced and when they should start to do exercise again).

Participants will be encouraged to continue to increase exercise to achieve Department of Health recommended levels of 60 minutes a day of a mixture of moderate/vigorous intensity aerobic with muscle strengthening activities on three days/week.

Prohibited: Advice on cognitive activity, discussion about the different types of cognitive activities. Instructions to record the cognitive activities.

Flexible*:* Assessment of range of movement. Advice on length of time at school (full days, half days, one lesson a day), support increasing time at school. Advice over

exams. Participants can be shown how to do stretches. They can also be offered a strengthening programme if this is one of their goals.
